# Mineral Oil as an Intestinal Lubricant

**Published:** 1916-11

**Authors:** 


### Mineral Oil as an Intestinal Lubricant.

It is probable that there is not 5 per cent of practicing physicians who have not had their attention called to the use of mineral oil as an intestinal lubricant, and, therefore, know nothing about it. Unfortunately, however, assuming that 95 per cent of the medical profession are practically or theoretically acquainted with such therapeutic use of mineral oil, it is a fact which could not in all probability be denied, that the great majority of these physicians have formed a more or less erroneous idea as to the modus operandi of the agent.

Many there are who express an opinion that the efficiency of mineral oil depends upon the high specific gravity. That this is a fallacy will be appreciated, if it will be remembered that the therapeutic object to be attained is not to get oil to pass through the intestinal canal in the shortest possible time. The heavier the oil, naturally the quicker it would tend to make its way to the anal outlet. Mineral oil does not possess physiological action to any degree. The restoration of loss of function to a bowel exhausted by useless attempts at peristalsis and impotent in the presence of masses of hard, unyielding and dry feces, is not brought about by "greasing it with oil." For protection of the intestinal mucous membrane, a thin coating of oil serves an excellent purpose, and the property which is responsible for the spreading of such an oil over the surface is not specific gravity, but what is popularly known as "spread."

The recovery of normal peristalsis in cases of constipation or obstipation is brought about by a softening of the fecal content of the bowel so that it can be easily molded and can adapt itself to changes in caliber or direction of the gut or make its way past obstructions, however produced. Consequently the properties of "penetration" and of "mix" are those which are most important in considering the action of mineral oil.

Many physicians have an idea that high viscosity is essential, forgetting that high viscosity does not mean good mixing or penetrating properties. Hence the physician who has used mineral oil haphazardly or with an idea that the higher its specific gravity and the greater its viscosity, the greater the results to be expected, has naturally scored probably as many failures—if not more—as he has successes.

Spread, mix and penetration are, therefore, the three most essential and important properties to be considered in making a selection from the many kinds of oil exploited to the medical profession—and to the public. Fifteen years' experience in the refining and adapting of Russian mineral oil for use as an intestinal lubricant has enabled its makers—McKesson & Robbins—to develop spread, mix and penetrating properties to the highest degree, so that in Liquid Albolene the physician may rest assured that it is a form of mineral oil which will practically guarantee him successful results, provided he uses it in the proper cases and *according to correct methods of administration.*

---

The advertising of McKesson & Robbins appears again in this issue after an absence of a few months. This good firm is finding that it pays to advertise in the TEXAS MEDICAL JOURNAL, and that it does not pay to stop the advertising. Therefore they will be regular advertising contributors hereafter.

---

A new advertisement appearing in this issue is that of the Nose-Ions Co. of New York. We commend a reading and consideration of their claims to physicians of the country.

---

The Louisiana Post-Graduate School of Medicine has doubled its advertising rate in this issue of the JOURNAL. This school is a Mecca for Southern physicians, and is constantly growing in attendance and in professional influence.

---

Vapo-Cresolene has been recognized as a standard product for many years, being especially destructive of diphtheria bacilli. The vapor is harmelss to the youngest child and yet has the germicidal value of carbolic acid. Physicians do not hesitate to use it regularly in their practice, and give strong endorsement to its worth in those cases for which it is recommended. See the advertising now appearing regularly in this JOURNAL.

